# Complete plastome of a subtropical tree *Eriobotrya malipoensis* (Rosaceae) in Yunnan

**DOI:** 10.1080/23802359.2019.1676680

**Published:** 2019-10-11

**Authors:** Shaohong Qu, Zhanghong Dong, Liyun Gao, Jian Xu, Zhenghai Sun, Peiyao Xin

**Affiliations:** a Key Laboratory of Forest Resources Conservation and Utilization in the Southwest Mountains of China Ministry of Education, Southwest Forestry University, Kunming, China;; b South and Southeast Asia Joint R&D Center of Economic Forest Full Industry Chain, Southwest Forestry University, Kunming, China;; c International Technologial Cooperation Base of High Effective Economic Forestry Cultirating, Southwest Forestry University, Kunming, China

**Keywords:** *Eriobotrya*, chloroplast, phylogenetic analyses

## Abstract

*Eriobotrya malipoensis* Kuan is an important wild woody evergreen tree within the genus *Eriobotrya* Lindl belonging the family Rosaceae. To better determine its phylogenetic location with respect to the other *Eriobotrya* species, the complete plastome of *E. malipoensis* was sequenced. The whole plastome is 159,313 bp in length, consisting of a pair of inverted repeat (IR) regions of 26,344 bp, one large single-copy (LSC) region of 87,270 bp, and one small single-copy (SSC) region of 19,355 bp. The overall G + C content of the whole plastome is 36.7%. Further, maximum likelihood phylogenetic analyse (TVM + F+R2 model) was conducted using 14 complete plastome of the Rosaceae. Our phylogeny supports the relationships: sisterhood of the *E. malipoensis* and *E. fragrans* Champ, flowed *E. japonica* Lindl.

*Eriobotrya malipoensis* Kuan is a narrowly distributed species at high altitudes in Southeast Yunnan of SW China. It was assigned to the genus *Eriobotrya* in the family Rosaceae (http://foc.iplant.cn/). *E. malipoensis* owns the largest leaves among the reported species in the genus *Eriobotrya* (Yang et al. 2017). Previous moleculae studies, using the RAPD, AFLP, internal transcribed spacer (ITS) and RAD sequencing technologies, reported close relationship between *E. malipoensis* and *E. japonica* (Li et al. 2009; Yang et al. 2009a, 2009b; Yang et al. 2017). Zhao et al. (2011), however, used ITS region to investigate sisterhood of *E. malipoensis* and *E. seguinii* rather than *E. japonica.* This raises the question, is there sister relationship between *E. malipoensis* and *E. japonica*? Here, we selected *E. malipoensis* to determine the entire plastid genome sequence.

The total genomic DNA was extracted from the fresh and healthy leaves of a single individual of *E. malipoensis*, which was collected from Malipo County (Yunnan, China; Long. 104.852021 E, Lat. 23.146254N, 1292 m), using the modified CTAB method (Shen et al. 2016). The voucher specimen was preserved in the Herbarium of Southwest Forestry University (Accession Number: SWFU-SY35055). The GetOrganelle software (Jin et al. 2018) was used to assemble the complete plastome of *E. malipoensis* with the publicly available plastome of *E. japonica* (GenBank accession number KT633951) as the reference (Huang 2019). Geneious R8.1.3 software (Biomatters Ltd, Auckland, New Zealand) was used for initial plastome annotation.

The plastome of *E. malipoensis* (LAU10002), with a length of 159,313 bp, was 176 bp and 27 bp larger than that of *E. japonica* (159,137 bp, KT633951) and *E. fragrans* (159,286 bp, LAU10001). It was also 844 bp and 926 bp smaller than that of *Pyrus ussuriensis* Maxim (160,157 bp, MK172841) and *Malus prattii* (Hemsl.) Schneid (160,239 bp, MH929090). The length of the inverted repeats (IRs), large single-copy (LSC), and small single-copy (SSC) regions of *E. malipoensis* was 26,344 bp, 87,270 bp, and 19,355 bp, respectively. The overall G + C content is 36.7% (LSC, 34.5%; SSC, 30.2%; IR, 42.7%). The plastid genome includes 112 unique genes, including 4 rRNA genes, 30 tRNA genes, and 78 protein-coding genes, of which 17 are duplicated in IR regions.

Furthermore, based on 13 published plastomes, we reconstructed a phylogenetic tree (Figure 1) to confirm the relationship between *E. malipoensis* and *E. fragrans* or *E. japonica*, with *Crataegus kansuensis* Wils (MF784433) as outgroup. Maximum likelihood (ML) phylogenetic analyses were performed based on TVM + F+R2 model in the iqtree version 1.6.7 programme with 1000 bootstrap replicates (Nguyen et al. 2015). The ML phylogenetic tree with 43-100% bootstrap values at each node supported the fact that *E. malipoensis* and *E. fragrans* instead of *E. japonica* were located in the same clade.

**Figure 1.**
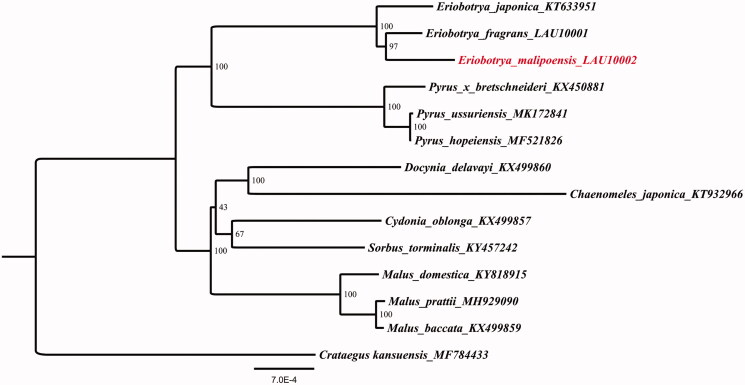
The ML phylogenetic tree for *E. malipoensis* based on other thirteen species (two in *Eriobotrya*, three in *Pyrus*, one in *Docynia*, one in *Chaenomeles*, one in *Cydonia*, one in *Sorbus*, three in *Malus*, and one in *Crataegus*) plastid genomes.

## Data Availability

The plastome data of the *E. malipoensis* will be submitted to Rosaceae Chloroplast Genome Database (https://lcgdb.wordpress.com). Accession numbers are LAU10002.
